# Evolving health information technology and the timely availability of visit diagnoses from ambulatory visits: A natural experiment in an integrated delivery system

**DOI:** 10.1186/1472-6947-9-35

**Published:** 2009-07-17

**Authors:** Naomi S Bardach, Jie Huang, Richard Brand, John Hsu

**Affiliations:** 1 Department of General Pediatrics, University of California, San Francisco, 3333 California St. Suite 245, San Francisco, CA 94118, USA; 2 Institute for Health Policy Studies, University of California, San Francisco, 3333 California St. Suite 265, San Francisco, CA 94118, USA; 3 Center for Health Policy Studies and Division of Research, Kaiser Permanente Northern California, 2000 Broadway, Oakland, CA 94612, USA; 4 Department of Epidemiology and Biostatistics, University of California, San Francisco, 185 Berry St. 5700, San Francisco, CA 94143-0560, USA; 5 School of Public Health, University of California, Berkeley, 50 University Hall, #7360, Berkeley, CA 94720-7360, USA

## Abstract

**Background:**

Health information technology (HIT) may improve health care quality and outcomes, in part by making information available in a timelier manner. However, there are few studies documenting the changes in timely availability of data with the use of a sophisticated electronic medical record (EMR), nor a description of how the timely availability of data might differ with different types of EMRs. We hypothesized that timely availability of data would improve with use of increasingly sophisticated forms of HIT.

**Methods:**

We used an historical observation design (2004–2006) using electronic data from office visits in an integrated delivery system with three types of HIT: Basic, Intermediate, and Advanced. We calculated the monthly percentage of visits using the various types of HIT for entry of visit diagnoses into the delivery system's electronic database, and the time between the visit and the availability of the visit diagnoses in the database.

**Results:**

In January 2004, when only Basic HIT was available, 10% of office visits had diagnoses entered on the same day as the visit and 90% within a week; 85% of office visits used paper forms for recording visit diagnoses, 16% used Basic at that time. By December 2006, 95% of all office visits had diagnoses available on the same day as the visit, when 98% of office visits used some form of HIT for entry of visit diagnoses (Advanced HIT for 67% of visits).

**Conclusion:**

Use of HIT systems is associated with dramatic increases in the timely availability of diagnostic information, though the effects may vary by sophistication of HIT system. Timely clinical data are critical for real-time population surveillance, and valuable for routine clinical care.

## Background

Health information technology (HIT) systems have the potential to improve health care quality and outcomes, in part by making important clinical data quickly available. [1-4] Timely information is critical at both the population and the individual patient levels. For instance, population surveillance for and monitoring of infectious disease outbreaks requires real time clinical information. [5-11] Similarly, coordination during natural disasters also benefits from timely exchange of clinical data, as seen during Hurricane Katrina in 2005.[12,13]

Newer electronic medical records (EMR) systems include many of the key capabilities for EMRs described by the Institute of Medicine in 2004,[14] such as electronic charting, computerized physician order entry, and clinical decision support. A study in Boston in 2001 demonstrated the accurate identification of lower respiratory tract infections with a comprehensive EMR, but did not explore the time to data availability associated with the EMR[15] and to our knowledge there are no studies demonstrating this relationship.

HIT also has potential for improving the care of individual patients, including improving communication between providers and allowing quicker access to information at the point of care by providing clinical documentation in a timely manner. [16-18] Prior studies have shown that data are often missing during clinical encounters within settings that predominantly rely on paper-based systems, and that the missing data may lead to adverse outcomes.[19,20] It is likely that one reason for missing data is delayed transfer of clinical information in a paper-based system; for instance, delays in documentation from a subspecialty visit arriving in the patient's record in the primary care office. While there are multiple descriptions of individual features of comprehensive EMRs,[21,22] the effect of EMRs on practitioner use of time [23-28] and quality of care delivered,[29] there have not been studies showing the changes in timely availability of clinical data associated with the implementation of a comprehensive EMR, nor a description of how the timely availability of data might differ with different types of EMRs.

In this study, we examined the association between the use of several types of EMRs in the outpatient setting, including a comprehensive EMR, and the timely availability of electronic data. We focused on the time to availability of visit diagnoses as recorded by clinicians either on paper visit diagnosis forms, or through two types of EMR systems in a large, prepaid, integrated delivery system (IDS) over a three-year period. The IDS's electronic databases captured all diagnoses during this time period; for diagnoses recorded on paper, data entry clerks transferred the information into the databases. The IDS sequentially introduced two new EMR systems for clinical care during this period, and a basic EMR system had already been in place, though clinicians infrequently used it for data entry. The natural experiment presented by the implementation of the various EMR systems allowed us to test our hypothesis that the timely availability of data would improve over time with increasing use of more sophisticated forms of HIT.

## Methods

### A. Setting

We conducted an historical review using electronic data from the Kaiser Permanente Northern California (KPNC) IDS from January 2004 through December 2006. The IDS provides comprehensive medical care to over three million members in Northern California. We focused on visit diagnosis data from outpatient clinic visits in internal medicine, family practice, pediatrics, and obstetrical-gynecological practices from January 2004–December 2006. We excluded visits to non-IDS sites. The KPNC institutional review board approved this study.

### B. Data

KPNC has had a number of functioning automated clinical databases for decades, though more limited HIT at the point-of-care; these databases permit the consistent capture of visit diagnoses and time when they were entered throughout the implementation of the new EMR systems, even though the data entry methods changed with the use of different HIT systems. These automated visit encounter databases also include information on membership status, individual socio-demographic characteristics, date and type of clinic visit, primary care physician, and medication prescriptions.

For this study, we abstracted data on the date and type of each clinic visit, clinician and patient identification codes, visit diagnoses as determined by the clinician, and date of diagnosis entry into the electronic encounters database, which was part of the legacy automated clinical databases, i.e., preceded the EMRs. Because clinicians or population managers could access the diagnostic information for population or point-of-care use only after entry into the electronic encounters database, we assessed the time between the visit and the entry of visit diagnosis into the database via one of the methods described below in the "Predictor" section. Throughout this paper, "visit diagnoses" refers to the diagnostic codes coded for each visit and entered into the electronic database. Diagnostic codes were standard across all KPNC sites, were SNOMED-CT (Systematized Nomenclature of Medicine – Clinical Terms) consistent, and were based on the International Statistical Classification of Diseases and Related Health Problems, version 9 (ICD-9).

### C. Predictor

**Figure 1:**
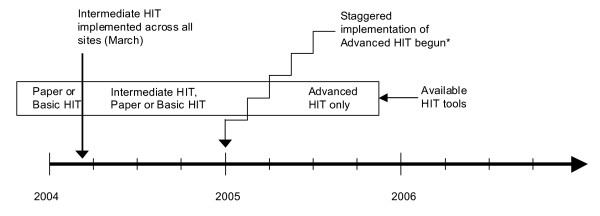
**Schematic of HIT implementation and use during study period**. *Advanced HIT implementation was staggered by medical center, and by team within medical center (estimated 3-week lag between teams in medical centers). Once Advanced HIT was implemented, no other interface was available for charting or entering diagnostic information.

Our main predictor was the method by which clinicians entered visit diagnoses (see Table 1 for summary of interfaces and Figure 1 for timeline of interface availability). The form for diagnostic code entry was similar across the three methods (paper, Basic, and Intermediate HIT systems), and resembled a standard billing form with ICD9-based diagnostic codes and checkboxes. The visit diagnoses entry processes were as follows:

**Table 1:** Charting systems characteristics

Charting system	EMR Features	Method of entry of visit diagnoses*
Paper	----	Paper form sent to a processing center, entered by clerk

Basic	Results viewing (labs), demographics	Basic interface, usually by clerks at the point of care working off of a paper form completed by the provider. Infrequently used method

Intermediate	Documentation, results viewing, medication ordering, referrals	Electronic version of the paper form, or a search bar within the EMR, entry by providers

Advanced	Documentation, radiology, laboratory, and medication ordering, results viewing, clinical decision support	Provider chooses ICD9-based diagnoses to create progress note for visit. These diagnoses are communicated to the electronic database after the provider completes chart

* Visit information from all four sources was linked to the same automated visit encounter database, the source of information for the study

EMR: electronic medical record

1) Paper: For visits involving paper medical records, clinicians completed a checkbox paper form at the end of the visit, which then went to a processing center where a clerk entered the diagnoses into the electronic database.

2) Basic HIT: At some sites using paper medical records, the diagnostic codes could be entered into the electronic database via the Basic HIT interface. Basic HIT, which was available at the start of the study, is a legacy mainframe system, and does not have charting features, but permits viewing of laboratory and radiology results.

3) In March 2004, Intermediate HIT became available across all sites simultaneously, but did not replace either the paper medical records or the Basic HIT system. Intermediate HIT was a web-based system that permitted free text entry of medical notes. Clinicians recorded diagnoses through this Intermediate interface (electronic version of the paper form for diagnostic coding or a search bar), which sent them directly to the IDS automated visit encounter databases. Intermediate HIT also had electronic referral and medication ordering features. Although Intermediate HIT was available beginning in March 2003, there was considerable variation in the onset and extent of its use from clinician to clinician.

4) Starting in the first quarter of 2005 (Figure 1), implementation of Advanced HIT began (also known as Kaiser Permanente HealthConnect or KPHC). Advanced HIT is an EpicCare^© ^system consisting of an integrated EMR with note templates, physician order entry, and results retrieval. Clinicians used the ICD9-based diagnostic codes to create the progress note, including the chief complaint and assessment sections. Once clinicians completed the visit note, the system sent the final visit diagnoses to the IDS's automated visit encounter databases. Thus, for Advanced HIT, entry of diagnostic codes was a proxy for completed visit documentation.

The Advanced HIT implementation was staggered between April 2005 and April 2008, across the 19 medical centers, and across clinical teams within each medical center. The Advanced HIT system completely replaced the paper medical record.

### D. Outcomes

To determine data availability, we measured the number of days between the date of the patient visit and the date of entry of the first diagnosis into the IDS's automated visit encounter database. Though diagnostic information may be entered into the electronic database at more than one point in time, 85.7%–99.8% of patient visits in any given month had all diagnoses entered into the system on the same day. In our sensitivity analyses, we found that all study findings were comparable whether we used the time of availability for the first or last diagnosis.

### E. Analyses

For each patient encounter in the electronic registration database, we recorded the method of entry of diagnostic information – paper form, Basic, Intermediate or Advanced HIT. For each month of the study period, for pooled office visits from all medical centers combined and in each medical center separately, we calculated: the percentage of visits using each type of HIT, and the percentage of visits with the first diagnosis entered into main electronic database by 0, 1, 4 or 7 days from the day of the visit.

Practitioner characteristics were available in the electronic databases for 86–87% of visits each year (age, gender, and training type). We obtained patient age, gender, and comorbidity score (DxCG) from the IDS's databases.[30,31] We also calculated the mean number of diagnoses per patient visit based on the diagnostic codes that had been entered into the electronic database.

In our examination of the relationship between HIT use and availability of diagnostic information, we did not adjust for possible changes in patient or practitioner mix over time, given the stability of patient and practitioner characteristics over time (Table 2). With our very large sample size and use of data from the entire target membership, standard statistical inference techniques were not relevant. Analyses were performed and data for measures were extracted using SAS 9.1.

## Results

During the study period, there were 29,490,302 total office visits. Table 2 presents numbers of visits annually and summary patient and practitioner characteristics. The annual number of visits increased slightly over time. On average, the patient and practitioner characteristics varied little over time.

**Figure 2:**
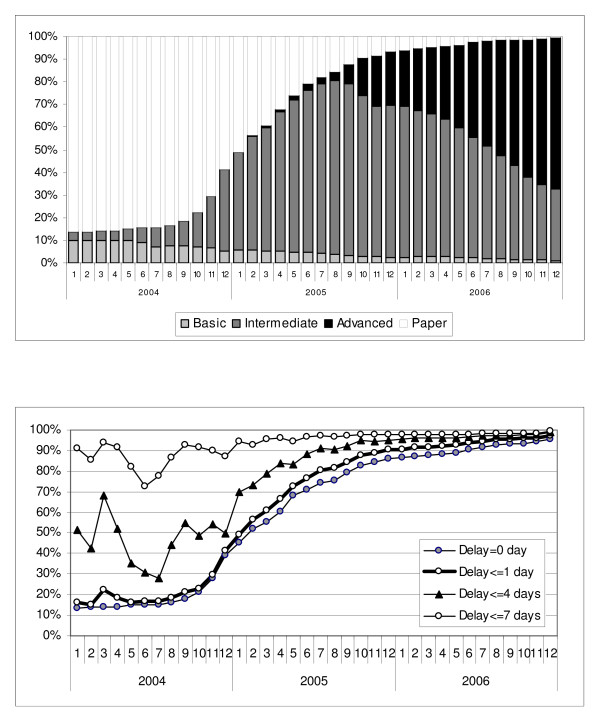
**All Office Visits' HIT Use and Time to Data Availability**. a. HIT use in all office visits. b. Percentage of all office visits with data available within 0, 1, 4, or 7 days.

**Figure 3:**
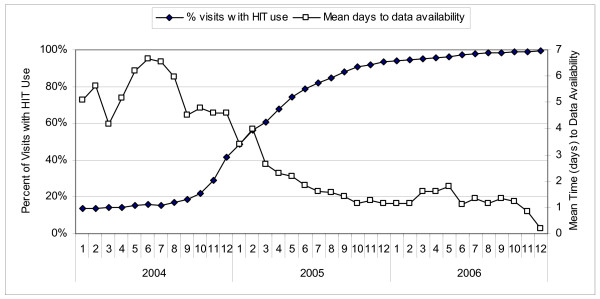
**Change in mean time to data availability with change in HIT use over time**.

For pooled visits across all medical centers and for each month of the study period, Figure 2a depicts the percentage with entry of diagnoses using paper or one of the HIT systems. The overall use of HIT increased with time, and as use of a more advanced form of HIT increased, use of the less advanced forms decreased. Figure 2b shows the increasing percentage of pooled visits with diagnostic data available on the same day (0 days), or by 1, 4, or 7 days after the patient visit, with the patterns of HIT use and data availability increasing similarly over time. In the beginning of the study period, corresponding to a time when most of the visits are paper based, only 13% of visits have diagnostic information available on the same day, with 91% of visits having diagnostic information available by the time a week has passed. In contrast, by the end of the study period, when HIT use had greatly increased (98% for all HIT types, and 67% Advanced HIT), 96% of office visits had diagnostic information available on the same day. Figure 3 summarizes the change in time to availability with the use of HIT throughout the IDS. The mean time to data availability throughout the study period ranged from 6.66 days to 0.2 days at the end of the period.

**Figure 4:**
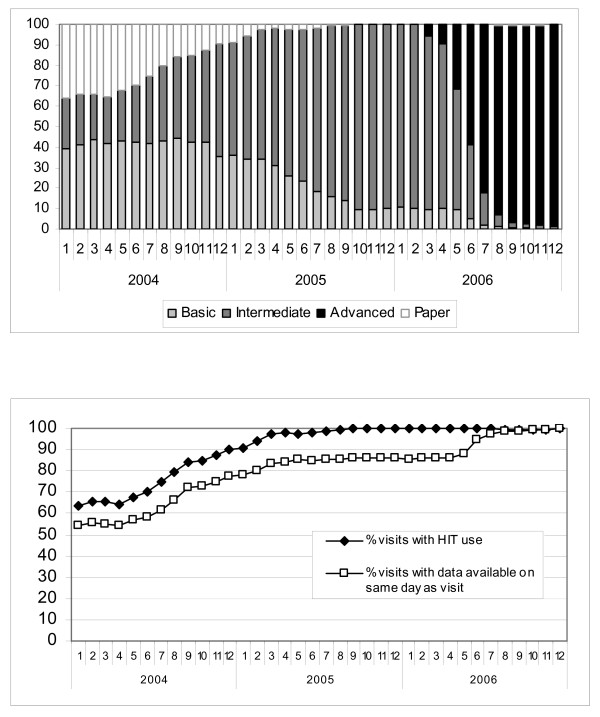
**Medical Center A increased use of Advanced HIT (Fig 4a) associated with >98% visits with data available on the same day as the visit (Fig 4b)**. a. HIT use in office visits at Medical Center A. b. Percent of office visits at Medical Center A with data available on the same day as the visit.

**Figure 5:**
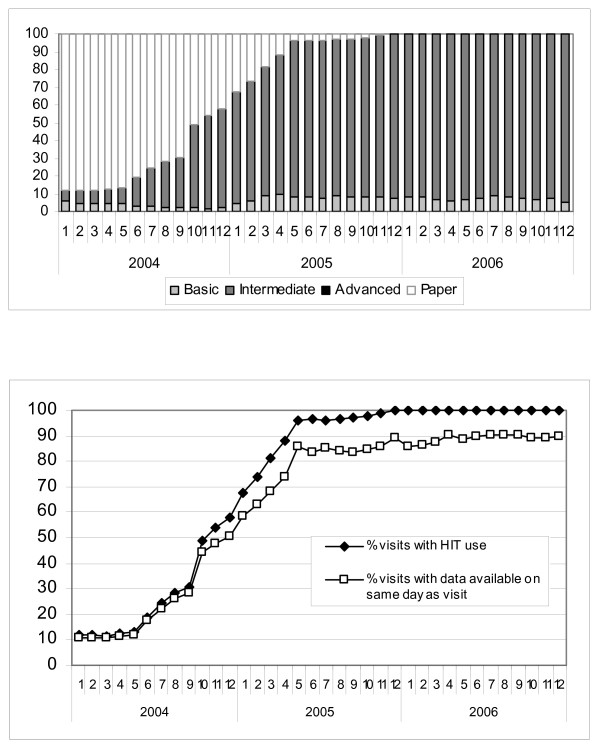
**At Medical Center B, Advanced HIT not implemented during study period (Fig 5a), and percentage of visits with data available on same day does not reach >91% (Fig 5b)**. a. HIT use in office visits at Medical Center B. b. Percent of office visits at Medical Center B with data available on the same day as the visit.

Across individual medical centers, the uptake of Intermediate and Advanced HIT differs, due to variable adoption of Intermediate HIT and the staggered implementation of Advanced HIT. Of note, in each medical center, as the use of more sophisticated HIT increases, there is a corresponding increase in the timely availability of diagnostic codes. Figures 4 and 5 depict illustrative patterns in two medical centers. In medical center A (Figure 4), gains in same-day availability of diagnostic data occurred with increasing use of Intermediate HIT but only progress to 100% with substantial use of Advanced HIT. In contrast, in medical center B (Figure 5), where Advanced HIT implementation had not begun by the end of the study period (December 2006), gains in same day availability of diagnostic data only rise to 90%, despite high use of the Intermediate HIT system (94% of visits). A similar pattern of greater rise in same day availability with use of Advanced HIT than with use of less sophisticated HIT occurs across the other 16 medical centers individually (data not shown).

## Discussion

In this study of almost 30 million visits within an integrated delivery system, there were dramatic increases in the percentage of office visits with diagnoses available within an electronic database on the same day as the visit during 2004–2006, corresponding to the implementation of increasingly sophisticated forms of HIT for office visit documentation.

Within any health system, there are multiple potential delays in visit documentation: 1) clinician delay, such as when clinical care takes precedence over documentation; 2) clinical information delay, such as when the diagnosis requires clinical information that is not immediately available, e.g., a laboratory result; and 3) systemic delays, such as prolonged processing time after the clinician finishes the record. The increase in timely availability of clinical data with HIT use likely results from decreased clinician delay and decreased systemic delays, since HIT allows for more swift entry of diagnoses or eliminates steps in the data capture process. One important advantage of an EMR over an entirely paper system is the decreased clinical information delay via swift access to laboratory and radiology results, for either the ordering clinician or another provider. This did not contribute in our study since results viewing was available throughout the study period, but may be a contributor in other systems that do not already have electronic access to laboratory values and radiology reports, particularly for care shifted between providers, when paper lab results may not be readily exchanged. Health care systems without such electronic transfer of laboratory, radiology, and consultation data might experience even more dramatic improvements in clinical data timeliness.

The problem of clinician delay may be exacerbated by the presence of sicker patients, which are more time intensive to care for and leave less time for documentation. We did not see any evidence for increase or decrease of acuity in the patient characteristics over time (age and number of diagnoses stayed had minimal variation), so it would be unlikely that a decrease in patient acuity would have contributed to the decrease in time to documentation. If the population had grown sicker during this time, then the effects of Advanced may be even more pronounced than they appear in our analysis.

The differential pattern of timely availability of diagnoses in individual medical centers reflecting the use of Advanced HIT at those centers (Figures 4 and 5) suggests that Advanced HIT may drive the timely entry of diagnoses more than Basic or Intermediate HIT. In most medical centers, once Advanced HIT was implemented for 80–90% of patient visits in the office setting, 99–100% of visits had documentation complete by the same day. While it is possible that Advanced HIT implementation increased medical center focus on timely documentation, the gains in timely data entry were coincident in each medical center with the uptake of Advanced HIT, and it is unlikely that every medical center had the same emphasis on timely documentation during the period of Advanced HIT implementation. The relationship between Advanced HIT and the more timely availability of information compared to Basic HIT or Intermediate HIT implies that different HIT functionality may lead to different effects on timely availability of clinical information, and that the systems that allow the most streamlined workflows may lead to the most timely information availability.

In other health care systems, individual providers may be motivated to complete diagnostic coding in order to get reimbursed in a more timely fashion. The results of our study, if applied to other medical providers using an EMR, suggest the possibility of timelier billing cycles. In contrast, KPNC is a prepaid, integrated delivery system, and while recording diagnostic codes is considered a part of standard clinical documentation for quality and organizational reasons, compensation is not dependent on entry of diagnostic codes. Therefore, improved billing cycles would be an unlikely driver in the increased timely availability of data demonstrated in this study.

In this study we did not find a change over time in the number of diagnoses for visits (Table 2). It has been hypothesized that an EMR would increase the number of diagnoses attributed to any patient, leading to improved data "completeness." A possible reason for improved completeness would include greater ease of documentation with an EMR.[32] It may be that the number of diagnostic codes entered did not change because the ones that were documented on paper records represented the complete clinical picture. It may be that there was no motivating factor for providers to increase the numbers of diagnoses charted in the EMR. We do not have information regarding this clinician behavior but it may be amenable to collection through qualitative or quantitative physician surveys.

**Table 2:** Patient and practitioner visit characteristics by setting annually

	2004	2005	2006
Number of visits	9.6 m	9.9 m	10 m

Patient characteristics			
Mean Age, years (SD)	43(24)	43(24)	43(24)
Female (%)	62	62	62
Mean comorbidity score (SD)	2.5 (3.7)	2.6(3.8)	2.7(4.0)
Mean diagnoses/visit	4.5	4.6	4.4

Practitioner characteristics			
Mean Age, years (SD)	46(9)	46(9)	46(9)
Female (%)	54	53	54
Training type (%)			
Internal medicine	31	31	30
Pediatrics	16	17	17
OB/Gyn	8.4	8.4	8.7
Family Medicine	7.1	7.1	8.8
Other	37	36	36

### A. Limitations

It is possible that some degree of change in the timely availability of diagnostic data results from changes in patient population or practitioner population over time rather than changes in HIT use over time. Though we did not adjust for possible confounders such as practitioner characteristics, patient characteristics, or seasonal variation, Table 2 demonstrates the stability of patient and practitioner characteristics over time. This provides evidence that any contribution they make to the change in availability of diagnostic data is likely to be small and not sufficient to explain the substantial increase in percentage of same-day availability of diagnostic data.

This study does not address the accuracy of the diagnoses entered. Errors in diagnostic accuracy may occur in two forms: diagnoses incorrectly attributed to the patient (e.g. assignation of hypertension when the patient has never been hypertensive); and diagnoses incorrectly attributed to the patient at the time of charting (e.g. anemia is recorded as a diagnosis even after it has resolved). Advanced HIT may facilitate the second type of error since it enables carryover of information from one visit to the next without a forcing mechanism to verify the information. The use of SNOMED codes, which allow for much more detailed specification of diagnoses than ICD-9 codes, may enable a more structured description of a visit, and therefore a more accurate one; demonstrating this was beyond the scope of this study but could be considered in the future.

The study occurred in a single, integrated delivery system. While the number of visits is quite large, findings may be specific to this patient population, practitioner population, or particular system, and may change with different HIT systems, or in non-integrated health care delivery systems. However, the Advanced HIT EpicCare^© ^system is commercially available, and therefore our findings provide information that may be useful to others considering implementing it in their own settings. Importantly, there was substantial time allotted to implementation of the Advanced HIT system to allow for adequate initial and ongoing clinician training, including policies such as decreased patient load for clinicians during the initial implementation at a site. This careful implementation and ongoing support may be more difficult to achieve in a smaller or non-integrated setting.

It was beyond the scope of this study to examine associations between changes in clinical outcomes and the improved timely availability of clinical information. However, documenting the effect the time to availability of clinical information has on the quality of care delivery and on individual outcomes would aid in quantifying the contributions EMRs make to improvements in overall health and we hope to examine these relationships in the future.

Finally, the study health system has collected electronic clinical data since well prior to the study period, so results may be different for organizations that have not previously collected electronic data. The change in timely availability with use of a HIT system may be more extreme for organizations that are still entirely paper-based.

### B. Implications

The current public health infrastructure is limited and suffers from a dearth of clinical information. Few public health officials receive routine, timely transfers of electronic data, have mechanisms to analyse the clinical data, monitor outbreaks, or even confirm other reports. Most public health localities rely instead on word of mouth reports, phone calls with clinicians, or in-person interviews.[8,33] Compounding this problem are the limited and often declining resources available for public and population health.

Electronic data captured by HIT systems offer tremendous promise in improving the availability of timely clinical information for disease surveillance, responses to potential outbreaks, and monitoring of actual outbreaks. For example, an electronic surveillance system implemented in laboratories in two Indiana counties led to a 29% increase in absolute number of *Shigella *species infections identified during an outbreak, and led to same day notification to the health department, decreased from an average lag time of 2.5 days from time of positive result to time of health department notification.[34] We have demonstrated that in an integrated delivery system using a comprehensive EMR, we can capture multiple different types of clinical diagnostic information, not just infectious disease entities.

In addition, HIT systems may improve quality of care by providing timely clinical information at the point of care. Patients with chronic disease require coordination of care across multiple practitioners, including primary care providers, specialists, care managers, pharmacists, and others. Relevant patient information often is not available at the time of the visit, which impedes clinical care.[20] A large survey of primary care physicians in Colorado, most using paper charts and a few using full electronic records, found physicians reported missing critical information in 13.6% of visits. The physicians also reported that the missing information resulted in potential delayed care or additional services for 59.5% of those visits.[20]

Patients who would have most likely benefited from the improved information availability in the KPNC system are those who re-present within one week of their visit. It has been shown in other settings that 6.4% of asthma patients seen for urgent care follow-up within one week.[35] We found previously that in 2002, KPNC members made 2.1 million visits for asthma (unpublished data), which implies up to ~133,000 repeat visits of an asthmatic within one week of initial presentation. Other common chronic diseases such as congestive heart failure, hypertension, and diabetes also may lead to repeat visits within a short time frame. It is these patients who will likely most benefit from HIT improvements in timely availability of information through processes such as medication management, coordination of care, and communication between providers.

## Conclusion

In an integrated delivery system, timely availability of clinical diagnostic information improved with use of increasingly sophisticated health information technology systems and with the elimination of paper-based charting. These findings were most prominent with increased use of an Advanced HIT system.

## Competing interests

The authors declare that they have no competing interests.

## Authors' contributions

NSB participated in the design of the study and drafted the manuscript. JH participated in the design of the study and data analysis. RB participated in the design of the study and statistical analysis. JTH participated in the design of the study. All authors read and approved the final manuscript.

## Pre-publication history

The pre-publication history for this paper can be accessed here:

http://www.biomedcentral.com/1472-6947/9/35/prepub
